# (*Z*)-Methyl 4-(1,3-benzothia­zol-2-yl­sulfan­yl)-2-(methoxy­imino)-3-oxo­butanoate

**DOI:** 10.1107/S1600536808040658

**Published:** 2008-12-06

**Authors:** Qian-Zhu Li, Bao-An Song, Song Yang, Yu-Guo Zheng, Qing-Qing Guo

**Affiliations:** a Center for Research and Development of Fine Chemicals, Guizhou University, Key Laboratory of Green Pesticide and Agricultural Bioengineering, Ministry of Education, Guiyang 550025, People’s Republic of China; b Department of Chemistry, Bijie University, Bijie 551700, People’s Republic of China

## Abstract

In the mol­ecular structure of the title compound, C_13_H_12_N_2_O_4_S_2_, there is a dihedral angle of 0.41 (13)° between the benzene and thia­zole rings. In the crystal, inversion dimers linked by two C—H⋯O inter­actions together with π–π stacking between the parallel benzene rings of adjacent mol­ecules [centroid–centroid distance = 3.673 (2) Å].

## Related literature

For general background to benzothia­zole derivatives and their biological activities, see: Bradshaw *et al.* (2008 ▶); Moharram (1990 ▶); Spillane *et al.* (2007 ▶).
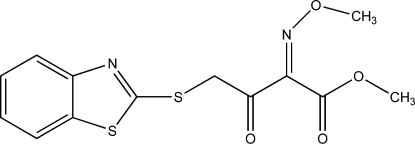


## Experimental

### 

#### Crystal data

C_13_H_12_N_2_O_4_S_2_
*M* *_r_* = 324.39
Triclinic, 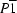

*a* = 8.019 (3) Å
*b* = 10.037 (4) Å
*c* = 10.662 (5) Å
α = 76.44 (2)°
β = 67.997 (14)°
γ = 74.964 (15)°
*V* = 759.3 (6) Å^3^
*Z* = 2
Mo *K*α radiation
μ = 0.37 mm^−1^
*T* = 293 (2) K
0.22 × 0.19 × 0.18 mm

#### Data collection

Bruker SMART CCD area-detector diffractometer
Absorption correction: multi-scan (*SADABS*; Bruker, 2002 ▶) *T*_min_ = 0.913, *T*_max_ = 0.939
7739 measured reflections
2620 independent reflections
2278 reflections with *I* > 2σ(*I*)
*R*_int_ = 0.020

#### Refinement

*R*[*F* ^2^ > 2σ(*F* ^2^)] = 0.034
*wR*(*F* ^2^) = 0.101
*S* = 1.10
2620 reflections
190 parameters
H-atom parameters constrained
Δρ_max_ = 0.23 e Å^−3^
Δρ_min_ = −0.27 e Å^−3^

### 

Data collection: *SMART* (Bruker, 2002 ▶); cell refinement: *SAINT* (Bruker, 2002 ▶); data reduction: *SAINT*; program(s) used to solve structure: *SHELXS97* (Sheldrick, 2008 ▶); program(s) used to refine structure: *SHELXL97* (Sheldrick, 2008 ▶); molecular graphics: *ORTEP-3 for Windows* (Farrugia, 1997 ▶); software used to prepare material for publication: *WinGX* (Farrugia, 1999 ▶).

## Supplementary Material

Crystal structure: contains datablocks global, I. DOI: 10.1107/S1600536808040658/at2688sup1.cif

Structure factors: contains datablocks I. DOI: 10.1107/S1600536808040658/at2688Isup2.hkl

Additional supplementary materials:  crystallographic information; 3D view; checkCIF report

## Figures and Tables

**Table 1:** Hydrogen-bond geometry (Å, °)

*D*—H⋯*A*	*D*—H	H⋯*A*	*D*⋯*A*	*D*—H⋯*A*
C3—H3⋯O1^i^	0.93	2.55	3.398 (3)	152

Symmetry code: (i) 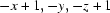.

**Fig. 1.**
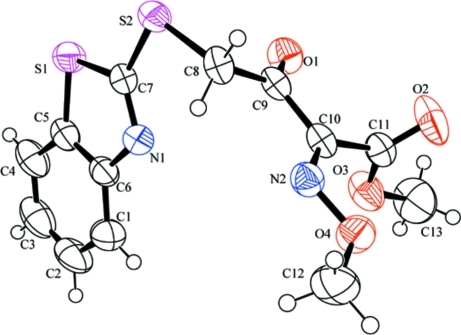
The molecular structure of (I) showing the atom-labelling scheme. Displacement ellipsoids are drawn at the 50% probability level.

## supplementary crystallographic information

### Comment

A wide range of biological activities have been attributed to compounds containing benzothiazole moiety such as anticancer (Bradshaw *et al.*, 2008), antibacterial (Moharram *et al.*, 1990) and cytotoxic activity (Spillane *et al.*, 2007), Herein we present the crystal structure of the title benzothiazole derivative (I).

The crystal structure of the title compound (I) is represented in Fig. 1. There is a dihedral angle of 0.41 (13)° between the benzene ring and thiazole ring. In the crystal structure, weak intermolecular C—H···O interactions (Table 1), together with π-π stacking between parallel benzene rings of adjacent molecules stabilize the packing, the centroid-to-centroid distance of two benzene rings is 3.673 (2)Å (symmetry codes: 1 - *x*,-*y*,1 - *z*).

### Experimental

A solution of (*Z*)-methyl-4-bromo-2-(methoxyimino)-3-oxobutanoate 30 ml (2.38 g, 0.01 mol) in methanol (10 ml) was added dropwise to a stirred solution of benzothiazole-2-thiol (1.67 g, 0.01 mol) and sodium hydroxide (0.40 g, 0.01 mol) in water (25 ml). The resulting mixture was stirred at room temperature for 3 h, the mixture was filtered and the residue was dissolved in 30 ml e thanol, Single crystals of (I) were obtained after several days.

### Refinement

H atoms were placed in calculated positions with C—H = 0.93–0.97 Å, and refined in riding mode with *U*_iso_(H) = 1.2–1.5 *U*_eq_(C).

### Crystal data

**Table:**

C_13_H_12_N_2_O_4_S_2_	*Z* = 2
*M**_r_* = 324.39	*F*(000) = 336
Triclinic, *P*1	*D*_x_ = 1.419 Mg m^−^^3^
Hall symbol: -P 1	Mo *K*α radiation, λ = 0.71073 Å
*a* = 8.019 (3) Å	Cell parameters from 2679 reflections
*b* = 10.037 (4) Å	θ = 2.1–25.0°
*c* = 10.662 (5) Å	µ = 0.37 mm^−^^1^
α = 76.44 (2)°	*T* = 293 K
β = 67.997 (14)°	Block, colourless
γ = 74.964 (15)°	0.22 × 0.19 × 0.18 mm
*V* = 759.3 (6) Å^3^	

### Data collection

**Table:**

Bruker SMART CCD area-detector diffractometer	2620 independent reflections
Radiation source: fine-focus sealed tube	2278 reflections with *I* > 2σ(*I*)
graphite	*R*_int_ = 0.020
φ and ω scans	θ_max_ = 25.0°, θ_min_ = 2.1°
Absorption correction: multi-scan (*SADABS*; Bruker, 2002)	*h* = −9→9
*T*_min_ = 0.913, *T*_max_ = 0.939	*k* = −11→11
7739 measured reflections	*l* = −12→12

### Refinement

**Table:**

Refinement on *F*^2^	Primary atom site location: structure-invariant direct methods
Least-squares matrix: full	Secondary atom site location: difference Fourier map
*R*[*F*^2^ > 2σ(*F*^2^)] = 0.034	Hydrogen site location: inferred from neighbouring sites
*wR*(*F*^2^) = 0.101	H-atom parameters constrained
*S* = 1.10	*w* = 1/[σ^2^(*F*_o_^2^) + (0.0551*P*)^2^ + 0.1857*P*] where *P* = (*F*_o_^2^ + 2*F*_c_^2^)/3
2620 reflections	(Δ/σ)_max_ < 0.001
190 parameters	Δρ_max_ = 0.23 e Å^−^^3^
0 restraints	Δρ_min_ = −0.26 e Å^−^^3^

### Special details

**Table:**

Geometry. All e.s.d.'s (except the e.s.d. in the dihedral angle between two l.s. planes) are estimated using the full covariance matrix. The cell e.s.d.'s are taken into account individually in the estimation of e.s.d.'s in distances, angles and torsion angles; correlations between e.s.d.'s in cell parameters are only used when they are defined by crystal symmetry. An approximate (isotropic) treatment of cell e.s.d.'s is used for estimating e.s.d.'s involving l.s. planes.
Refinement. Refinement of *F*^2^ against ALL reflections. The weighted *R*-factor *wR* and goodness of fit *S* are based on *F*^2^, conventional *R*-factors *R* are based on *F*, with *F* set to zero for negative *F*^2^. The threshold expression of *F*^2^ > σ(*F*^2^) is used only for calculating *R*-factors(gt) *etc*. and is not relevant to the choice of reflections for refinement. *R*-factors based on *F*^2^ are statistically about twice as large as those based on *F*, and *R*- factors based on ALL data will be even larger.

### Fractional atomic coordinates and isotropic or equivalent isotropic displacement parameters (Å^2^)

**Table:**

	*x*	*y*	*z*	*U*_iso_*/*U*_eq_	
C1	0.2301 (4)	0.0381 (3)	0.4741 (3)	0.0665 (7)	
H1	0.1206	0.0298	0.5455	0.080*	
C2	0.3264 (5)	−0.0698 (2)	0.3972 (3)	0.0788 (9)	
H2	0.2803	−0.1514	0.4185	0.095*	
C3	0.4890 (4)	−0.0581 (3)	0.2898 (3)	0.0735 (8)	
H3	0.5497	−0.1320	0.2407	0.088*	
C4	0.5617 (4)	0.0593 (3)	0.2549 (3)	0.0635 (6)	
H4	0.6707	0.0670	0.1829	0.076*	
C5	0.4664 (3)	0.1679 (2)	0.3314 (2)	0.0472 (5)	
C6	0.3029 (3)	0.1583 (2)	0.4407 (2)	0.0452 (5)	
C7	0.3251 (3)	0.3681 (2)	0.45184 (18)	0.0387 (4)	
C8	0.0771 (3)	0.5086 (2)	0.65173 (19)	0.0436 (5)	
H8A	0.0138	0.5988	0.6798	0.052*	
H8B	−0.0068	0.4706	0.6302	0.052*	
C9	0.1277 (3)	0.41269 (19)	0.76915 (19)	0.0399 (4)	
C10	−0.0218 (3)	0.3515 (2)	0.88308 (19)	0.0403 (4)	
C11	0.0216 (3)	0.2738 (2)	1.0097 (2)	0.0443 (5)	
C12	−0.4696 (3)	0.3219 (3)	0.9528 (3)	0.0694 (7)	
H12A	−0.5552	0.2765	1.0303	0.104*	
H12B	−0.4454	0.2809	0.8734	0.104*	
H12C	−0.5204	0.4195	0.9369	0.104*	
C13	0.1207 (5)	0.0538 (3)	1.1217 (3)	0.0890 (9)	
H13A	0.1554	−0.0424	1.1090	0.134*	
H13B	0.0174	0.0646	1.2040	0.134*	
H13C	0.2215	0.0828	1.1287	0.134*	
N1	0.2241 (2)	0.27441 (17)	0.50850 (16)	0.0448 (4)	
N2	−0.1769 (2)	0.36936 (18)	0.86710 (16)	0.0451 (4)	
O1	0.28034 (19)	0.38332 (16)	0.77643 (15)	0.0530 (4)	
O2	0.0143 (3)	0.32938 (19)	1.09837 (17)	0.0774 (5)	
O3	0.0721 (2)	0.13890 (16)	1.00578 (16)	0.0638 (4)	
O4	−0.30078 (19)	0.30477 (17)	0.97942 (15)	0.0568 (4)	
S1	0.52463 (7)	0.32799 (6)	0.31355 (5)	0.05263 (18)	
S2	0.27235 (7)	0.53273 (5)	0.50010 (5)	0.04578 (17)	

### Atomic displacement parameters (Å^2^)

**Table:**

	*U*^11^	*U*^22^	*U*^33^	*U*^12^	*U*^13^	*U*^23^
C1	0.0938 (19)	0.0544 (14)	0.0641 (14)	−0.0322 (13)	−0.0349 (13)	0.0011 (11)
C2	0.138 (3)	0.0375 (13)	0.093 (2)	−0.0260 (15)	−0.074 (2)	0.0004 (13)
C3	0.103 (2)	0.0500 (14)	0.0869 (19)	0.0087 (14)	−0.0594 (18)	−0.0267 (13)
C4	0.0734 (16)	0.0570 (14)	0.0702 (15)	0.0041 (12)	−0.0340 (13)	−0.0302 (12)
C5	0.0562 (12)	0.0444 (11)	0.0480 (11)	−0.0042 (9)	−0.0252 (10)	−0.0131 (9)
C6	0.0596 (12)	0.0385 (11)	0.0474 (11)	−0.0108 (9)	−0.0292 (10)	−0.0048 (8)
C7	0.0414 (10)	0.0418 (10)	0.0358 (9)	−0.0071 (8)	−0.0154 (8)	−0.0082 (8)
C8	0.0432 (11)	0.0490 (11)	0.0406 (10)	0.0008 (9)	−0.0174 (8)	−0.0155 (8)
C9	0.0420 (11)	0.0403 (10)	0.0438 (10)	0.0002 (8)	−0.0203 (8)	−0.0174 (8)
C10	0.0438 (10)	0.0413 (10)	0.0424 (10)	−0.0005 (8)	−0.0213 (8)	−0.0155 (8)
C11	0.0440 (11)	0.0488 (12)	0.0439 (10)	−0.0041 (9)	−0.0188 (9)	−0.0127 (9)
C12	0.0513 (13)	0.0842 (18)	0.0843 (17)	−0.0193 (12)	−0.0342 (13)	−0.0082 (14)
C13	0.127 (3)	0.0679 (18)	0.0844 (19)	−0.0117 (17)	−0.068 (2)	0.0130 (15)
N1	0.0484 (9)	0.0457 (10)	0.0433 (9)	−0.0136 (8)	−0.0150 (7)	−0.0077 (7)
N2	0.0446 (9)	0.0497 (10)	0.0449 (9)	−0.0089 (7)	−0.0185 (7)	−0.0088 (7)
O1	0.0438 (8)	0.0620 (9)	0.0594 (9)	−0.0054 (7)	−0.0272 (7)	−0.0094 (7)
O2	0.1180 (15)	0.0696 (11)	0.0605 (10)	0.0034 (10)	−0.0516 (10)	−0.0270 (9)
O3	0.0931 (12)	0.0467 (9)	0.0642 (10)	−0.0046 (8)	−0.0473 (9)	−0.0069 (7)
O4	0.0463 (8)	0.0745 (11)	0.0546 (9)	−0.0184 (7)	−0.0238 (7)	0.0000 (7)
S1	0.0523 (3)	0.0552 (4)	0.0485 (3)	−0.0167 (3)	−0.0037 (2)	−0.0193 (2)
S2	0.0515 (3)	0.0413 (3)	0.0472 (3)	−0.0109 (2)	−0.0144 (2)	−0.0131 (2)

### Geometric parameters (Å, °)

**Table:**

C1—C6	1.389 (3)	C8—H8A	0.9700
C1—C2	1.397 (4)	C8—H8B	0.9700
C1—H1	0.9300	C9—O1	1.210 (2)
C2—C3	1.387 (4)	C9—C10	1.493 (3)
C2—H2	0.9300	C10—N2	1.279 (2)
C3—C4	1.362 (4)	C10—C11	1.506 (3)
C3—H3	0.9300	C11—O2	1.182 (2)
C4—C5	1.398 (3)	C11—O3	1.315 (3)
C4—H4	0.9300	C12—O4	1.445 (3)
C5—C6	1.398 (3)	C12—H12A	0.9600
C5—S1	1.736 (2)	C12—H12B	0.9600
C6—N1	1.397 (3)	C12—H12C	0.9600
C7—N1	1.291 (2)	C13—O3	1.450 (3)
C7—S2	1.745 (2)	C13—H13A	0.9600
C7—S1	1.753 (2)	C13—H13B	0.9600
C8—C9	1.507 (3)	C13—H13C	0.9600
C8—S2	1.800 (2)	N2—O4	1.386 (2)
			
C6—C1—C2	118.0 (3)	O1—C9—C10	118.71 (17)
C6—C1—H1	121.0	O1—C9—C8	124.10 (19)
C2—C1—H1	121.0	C10—C9—C8	117.19 (16)
C3—C2—C1	121.5 (2)	N2—C10—C9	118.18 (17)
C3—C2—H2	119.3	N2—C10—C11	124.72 (18)
C1—C2—H2	119.3	C9—C10—C11	117.11 (16)
C4—C3—C2	121.3 (2)	O2—C11—O3	125.40 (19)
C4—C3—H3	119.3	O2—C11—C10	123.41 (19)
C2—C3—H3	119.3	O3—C11—C10	111.17 (16)
C3—C4—C5	117.6 (3)	O4—C12—H12A	109.5
C3—C4—H4	121.2	O4—C12—H12B	109.5
C5—C4—H4	121.2	H12A—C12—H12B	109.5
C6—C5—C4	122.2 (2)	O4—C12—H12C	109.5
C6—C5—S1	109.43 (15)	H12A—C12—H12C	109.5
C4—C5—S1	128.4 (2)	H12B—C12—H12C	109.5
C1—C6—N1	124.8 (2)	O3—C13—H13A	109.5
C1—C6—C5	119.4 (2)	O3—C13—H13B	109.5
N1—C6—C5	115.76 (18)	H13A—C13—H13B	109.5
N1—C7—S2	124.48 (15)	O3—C13—H13C	109.5
N1—C7—S1	117.24 (15)	H13A—C13—H13C	109.5
S2—C7—S1	118.25 (11)	H13B—C13—H13C	109.5
C9—C8—S2	113.07 (14)	C7—N1—C6	109.26 (17)
C9—C8—H8A	109.0	C10—N2—O4	111.87 (15)
S2—C8—H8A	109.0	C11—O3—C13	115.94 (19)
C9—C8—H8B	109.0	N2—O4—C12	109.46 (16)
S2—C8—H8B	109.0	C5—S1—C7	88.31 (10)
H8A—C8—H8B	107.8	C7—S2—C8	98.95 (10)

### Hydrogen-bond geometry (Å, °)

**Table:**

*D*—H···*A*	*D*—H	H···*A*	*D*···*A*	*D*—H···*A*
C3—H3···O1^i^	0.93	2.55	3.398 (3)	152

Symmetry codes: (i) −*x*+1, −*y*, −*z*+1.
